# Placental pathology of the third trimester pregnant women from COVID-19

**DOI:** 10.1186/s13000-021-01067-6

**Published:** 2021-01-14

**Authors:** Likun Gao, Jiacai Ren, Li Xu, Xiaokang Ke, Lin Xiong, Xiaoli Tian, Cuifang Fan, Honglin Yan, Jingping Yuan

**Affiliations:** 1 Department of Pathology, Renmin Hospital of Wuhan University, 99 Ziyang Road, Wuchang District, Wuhan, 430060 Hubei Province People’s Republic of China; 2 Department of Obstetrics and Gynecology, Renmin Hospital of Wuhan University, Wuhan City, 430060 Hubei Province People’s Republic of China

**Keywords:** Severe acute respiratory syndrome coronavirus 2 (SARS-CoV-2), Placenta pathology, The third trimester pregnancy, Hofbauer cells, Syncytial knots

## Abstract

**Aims:**

To explore the clinical characteristics and placental pathological changes of pregnant women with 2019 novel coronavirus (CoV) disease (COVID-19) in the third trimester, and to assess the possibility of vertical transmission.

**Methods and results:**

The placenta tissues were evaluated by using immunohistochemistry for inflammatory cells and Hofbauer cells, and using severe acute respiratory syndrome (SARS) CoV-2 RNA Fluorescence In-Situ Hybridization (FISH) and SARS-CoV-2 spike protein immunofluorescence (IF) double staining. All eight placentas from the third trimester pregnancy women were studied. All patients were cured, no clinical or serological evidence pointed to vertical transmission of SARS-CoV-2. Features of maternal vascular malperfusion (MVM) such as increased syncytial knots were present in all 8 cases (8/8), and increased focal perivillous fibrin depositions were presented in 7 cases (7/8). No significate chronic histiocytic intervillositis was noted in the placenta. The number of macrophages and inflammatory cells such as T cells, B cells and plasma cells in the placental villous was not significantly increased in all cases. Moreover, all of eight cases demonstrated negative results by FISH using a SARS-CoV-2 virus RNA probe and by IF using a monoclonal antibody against SARS-CoV-2 spike protein.

**Conclusions:**

We found no evidence of vertical transmission and adverse maternal-fetal outcomes in the placentas of third trimester COVID-19 pregnancy women, which provided further information for the clinical management of those women in the third trimester. However, further studies are still needed for patients with infections in different stage of gestation, especially in first and second trimester.

## Introduction

Since December 2019, the highly contagious 2019 novel coronavirus (CoV) disease (COVID-19) [1, 2] has affected more than 76.84 million persons and the number of death cases has reached more than 1.69 million worldwide, as of December 21, 2020. Most of COVID-19 patients showed mild upper respiratory infection symptom, but occasional might progress to severe illness even respiratory failure in some individuals [3, 4]. Contrasted to the overall population, pregnant women were a special group of significantly higher risk of viral pneumonia as the unique ‘immunological’ condition and changes in lung function during pregnancy [5–7], and intrauterine infection is one of the most serious complications of viral diseases during pregnancy. Since the evidence of coronaviruses infection such as severe acute respiratory syndrome (SARS) and Middle East respiratory syndrome (MERS) showed severe adverse pregnancy outcomes [8–11], the effects of the severe acute respiratory syndrome coronavirus 2 (SARS-COV-2) infection on the pregnant women and their fetus has caught worldwide attention. The majority of newborns delivered by pregnant women infected with SARS-COV-2 were negative for the virus, but a few tested positive for the virus infection. It is important to determine whether the intrauterine transmission of SARS-COV-2 has occurred and its development mechanisms [12–15]. In order to protect the fetus from various pathogens that may be infected during pregnancy, the placenta plays an important role as a natural barrier [16]. Recently, several cases of the SARS-COV-2 invasion of the placenta in the second and third trimester pregnant women have been confirmed clearly [13, 17–19], which suggested that transplacental transmission could occur. In addition, the pathology criteria for the diagnosis of intrauterine transplacental infection have been published [20], but the number of cases targeted for such studies is still limited, and the specific mechanism of the SARS-COV-2 invasion of the placenta in late pregnancy women is still not completely clear. Therefore, to investigate the above question, further studies is needed to examine the structure of the placenta and to explore the role of the placenta in the vertical transmission mechanism of COVID-19 pregnancy women.

Notably, placental pathology can provide vital information on the change of the human placenta structure and the mechanisms of maternal-fetal transmission for pathogens infection [21], as well as the effects of the organisms on the placenta to the virus infection such as the inflammatory response change, necrosis, hemorrhage, or vascular disease [22]. In addition, recent studies have shown that placenta has a unique capacity to prevent expansion of the virus and transmission to the fetus [16, 23, 24]. So, focusing on placenta feature may contribute to understand the effect of virus infection on maternal and fetus safety. As the few existing studies are not only limited to the number of patients, but also to simple microscopic observations, it is very necessary to further study the placenta of the late pregnancy women with SARS-COV-2 infection from the three levels of histology, immunohistochemistry, and molecular genetics.

In this study, we collected placenta tissues from 8 cases of pregnant women with COVID-19 in their third trimester. We aimed to analyze the clinical characteristics of SARS-COV-2 infected pregnant women and their neonates, and to detail the placental pathological changes by histology observations and immunohistochemical detection of inflammatory cells and fetal-derived placental macrophages (Hofbauer cells) in the placenta, and to determine the occurrence of SARS-CoV-2 infection of placentas by Fluorescence In-Situ Hybridization (FISH). Our study tried to establish corresponding clinicopathological links, evaluate the possibility of the intrauterine vertical transmission and provide a basis for the optimal management of such pregnant women.

## Materials and methods

### Clinical data collection

This study was conducted following expedited instigutional review board approval. Pregnant women with COVID-19 confirmed by Renmin Hospital of Wuhan University between January 30 and April 23, 2020, were included. The main contents in clues corresponding clinical history of the mother and infant, laboratory test results and chest CT scan data were abstracted from the electronic medical record system. In addition, this study has gathered the information on obstetric and neonatal outcomes. Major medical complications also be identified. Finally, SARS-CoV-2 RNA real-time reverse transcription polymerase chain reaction (RT-PCR) was performed in all neonates by pharyngeal swab to confirm whether there was evidence of perinatal transmission.

### Histopathological examination

According to the recommendation of SARS-CoV-2 surgical specimen specification fixation, all fresh placentas of COVID-19 pregnant women in third trimester were collected and delivered to the Department of Pathology for comprehensive pathological examination after standard clinical precautions. The standard examination protocol mainly consisted of fixation in 3.7% formaldehyde solution, sectioning, and careful examination the cut surface. Sections submitted included extraplacental membranes, umbilical cord, placenta and representative sampling of any lesions present. After paraffin embedding, routine H&E staining protocol was performed. The section thickness was 4 μm. All cases were reviewed by 2 pathologists to confirm the diagnosis.

### Immunohistochemical studies

Briefly, the placenta samples of all 8 patients were fixed with formalin, taken and prepared into paraffin blocks according to the standard procedure. Then the blocks were cut into 4 μm thick sections for the next immunohistochemistry (IHC) operation. IHC staining was performed in a DAKO Autostainer system (DAKO, Glostrup, Denmark) according to the manufacturer’s protocol instructions. The list of primary antibodies was as follows: anti-CD3, anti-CD20, anti-CD163, anti-CD68, anti-CD138, which were all purchased from DAKO (Glostrup, Denmark). In addition, 3 cases of paraffin-embedded placenta tissues in the third trimester without abnormal histology served as controls.

### SARS-CoV-2 RNA fluorescence in-situ hybridization and SARS-CoV-2 spike protein immunofluorescence double staining for placenta tissue

Firstly, immunofluorescence (IF) with an antibody against SARS-CoV-2 spike protein was conducted in accordance with the manufacturer’s protocol (Servicebio, Wuhan, China)**.** Secondly, Fluorescence In-Situ Hybridization (FISH) was performed for detecting the genomic RNA of SARS-CoV-2 virus from placenta formalin-fixed, paraffin-embedded (FFPE) tissues in accordance with the manufacturer’s protocol (Servicebio, Wuhan, China). The RNA probe oligo nucleotides which carrys one CY3 fluorophore targeting specific areas of SARS-CoV-2 virus and contains 22 nucleotides were designed and synthesized by Servicebio. The probe sequence is 5′-CY3-CCGUC UGCGG UAUGU GGAAA GGUUA UGG-3′. Briefly, tissue sections were handled by the FISH standard examination protocol which consisted of deparaffinizing, washing by ethanol, and blocking endogenous peroxidase. To induce epitope retrieval, the sections were heated in buffer. Then all sections were digested by proteinase and incubated with probe over night at 50 °C. After washing, FISH signals in cells were analyzed by fluorescence microscopy. A paraffin-embedded colon tissue with SARS-CoV-2 infection from another COVID-19 patient served as a positive control for detection of SARS-CoV-2 mRNA expression [25]. The results were viewed and visualized by an Olympus Eclipse 55i microscope (Olympus, Tokyo, Japan).

## Result

### Clinical features of all 8 pregnant women with SARS-CoV-2 infection

During the study period, there was a total of 8 pregnant women who were hospitalized for COVID-19 in Renmin Hospital of Wuhan University in Wuhan, Table 1 summarized detailed clinical relevant data. Eight patients ranged in age from 25 to 40 years. The stage of gestation at admission ranged between 33 weeks to 40 weeks plus 1 day. Four patients had mild symptoms related to COVID-19 pneumonia. None of the 8 patients showed symptoms of high fever (body temperature > 39 °C). Only one patient had continuous fever for 3 days before delivery (case 8, temperature was 38.5 °C), but no fever after delivery. One patient had cough (case 3). RT-PCR showed positivity for SARS-CoV-2 RNA in the nasopharyngeal (NP) swabs of all patients in their third trimester. The major complications of all 8 patients were as follows: anemia (Case 1 and 3), hypertension and low amniotic fluid (Case 2), pericardial effusion (Case 3), thrombocytopenia (Case 3), glomerulonephritis and hypothyroidism (Case 4).

**Table 1:** Demographical and clinical characteristics of the recovered COVID-19 infected pregnant women

Characteristics	patient							
	1	2	3	4	5	6	7	8
Date of admission (month-day)	4–18	4–6	3–24	3–24	3–18	4–23	1–30	1–31
Age at admission (year)	34	25	27	37	32	32	29	40
Gestational age (weeks) on admission	39^+ 2^	39^+ 5^	39^+ 1^	33^+ 1^	40^+ 1^	39^+ 5^	37^+ 1^	37^+ 6^
Recovery	+	+	+	+	+	+	+	+
Complications	AN	HT LAF	AN;PE TB	GR HT	–	–	–	–
Clinical classification Asymptomatic	A	A	M	M	A	A	M	M
**signs and symptoms**								
Fever on admission	–	–	–	37.4	–	–	38.5 (3 days)	–
Post-partum fever	–	–	–	–	–	–	–	–
Myalgia	–	–	–	–	–	–	–	–
Malaise	–	–	–	–	–	–	–	–
Cough	–	–	+	–	–	–	–	–
Chest pain	–	–	–	–	–	–	–	–
Sore throat /muscle pains	–	–	–	–	–	–	–	–
Diarrhoea	–	–	–	–	–	–	–	–
abdominal pains	–	–	–	–	–	–	–	–
chills	–	–	–	–	–	–	–	–
**Laboratory characteristics**								
White blood cell count(× 10^9^)	3.45	10.89	9.84	7.41	NA	6.33	NA	19.8
lymphocyte count(× 10^9^)	0.93	1.72	1.15	1.29	NA	0.96	NA	0.89
Elevated C-reactive protein concentration (mg/L)	<5.0	<5.0	27.5	<5.0	18.9	<5.0	NA	NA
ALT(U/L)	9	11	NA	14	NA	7	NA	NA
AST(U/L)	15	23	NA	17	NA	14	NA	NA
SARS-CoV-2 quantitative RT-PCR	+	+	+	+	+	+	+	+
CT typical evidence of viral infection pneumonia	+	–	+	+	+	–	–	+
**Delivery**								
Method of delivery	CS	CS	CS	CS	CS	SD	CS	CS
Indication for delivery	CoV	CoV	CoV	CoV	CoV	/	CoV	CoV
Date of delivery (month-day)	4–18	4–6	3–25	3–21	3–18	4–23	1–30	1–31
Diagnosis to termination of pregnancy (day)	0	0	1	5	0	0	0	0
**Newborn infants**								
Apgar score(1 min)	8	9	9	9	9	9	9	9
Apgar score(5 min)	10	10	10	10	10	10	10	10
Neonatal weight (kg)	3.55	3.3	3.23	2.17	3.35	3.45	3.1	2.95
Neonatal asphyxia	–	–	–	–	–	–	–	–
Neonatal death	–	–	–	–	–	–	–	–
Neonatal birth defect	+^#^	–	–	–	–	–	–	–
SARS-CoV-2 quantitative RT-PCR for infant	–	–	–	–	–	–	–	–

/: not applicable or data missing;+: yes or positive; −: no or negative

Clinical classification: asymptomatic (A), mild syndrome (M); *AN* anemia, *HT* hypertension, *LAF* low amniotic fluid, *PE* pericardial effusion, *TB* thrombocytopenia, *GR* glomerulonephritis, *HT* hypothyroidism, *IA* Induced abortion, *NA* not acquired, *CS* Caesarean section, *SD* spontaneous delivery, *SR* self-request, CoV:COVID-19 pneumonia; #: Case 1 had a 2 × 2 cm defect on the top of the head at birth

Laboratory examination showed that some patients with COVID-19 had slightly elevated C-reactive protein (2/8,>5.0 mg/L). Additionally, no one presented leukopenia and lymphopenia, and all patients had normal concentrations of alanine aminotransaminase (ALT) and aspartate aminotransferase (AST). All 8 patients underwent chest CT scan. Five patients (Case 1, 3, 4, 5 and 8) had multiple patchy ground-glass density shadows in both lungs, which is the typical manifestation of chest CT images of SARS-CoV-2 lung infection.

Seven patients were delivered by cesarean section, but one patient (Case 6) by natural delivery. The Apgar score of all newborns were 8 or 9 at 1 min and 10 at 5 min at birth, and underwent SARS-CoV-2 pharyngeal swab nucleic acid testing, all of which were negative for SARS-CoV-2 infection. Case 1 had a 2 × 2 cm defect on the top of the head at birth. Up to date, all of the mothers recovered well and were discharged home asymptomatic, no clinical or serological evidence pointed to vertical transmission of SARS-CoV-2.

### Pathological findings in the placentas of the pregnant women with SARS-CoV-2 infection

**Fig. 1:**
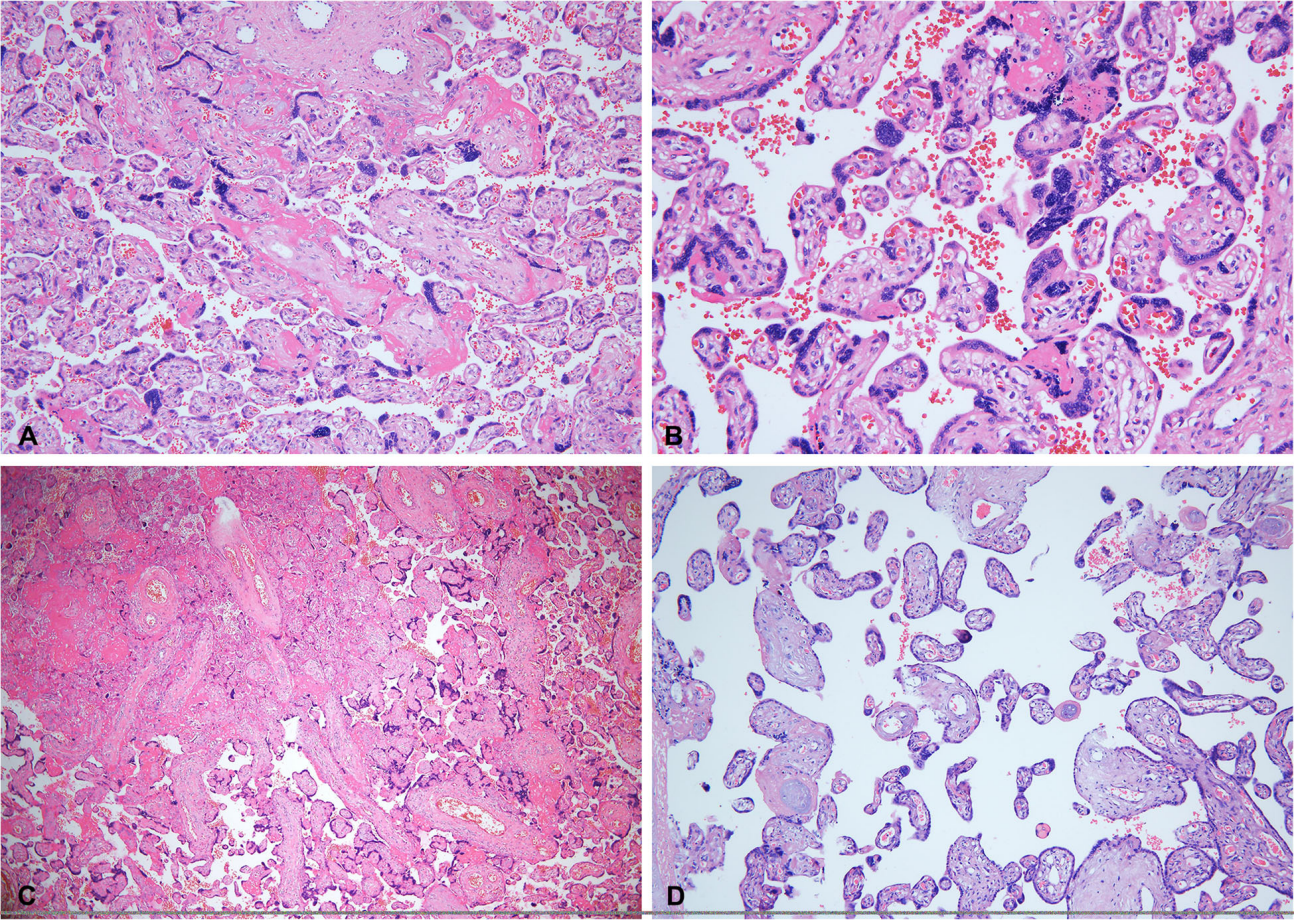
Microscopy of the placentas. **a**, Low power view with the chorionic plateof case 3 (39^+ 1^ weeks gestation). Increased focal perivillous fibrin depositions and increased syncytial knots were presented (H&E, original magnifications× 100). **b**, Close up view of increased syncytial knots in the terminal villi with the chorionic plate of case 3 (H&E, original magnifications× 200). **c**, Low power view with the chorionic plate of case 2(39^+ 5^ weeks gestation). Central placental infarct was presented (H&E, original magnifications× 40). **d**, Low power view with the chorionic plate of case 8(37^+ 6^ weeks gestation). Distal villous hypoplasia was presented (H&E, original magnifications× 100)

All cases were intact placenta. In general, 8 cases of intact placenta tissues were all sponge-like and dark red, grossly normal from the appearance. Under the microscopic examination of the placental disc, as shown in Table 2, only 1 case (case 8) showed edema in the villous stroma and mild acute intervillositis, the inflammatory infiltration was foci-like and consisted of several neutrophils and scanty histiocytes, no trophoblast necrosis and massive perivillous fibrin deposition was found. 2 cases showed chronic plasma cell deciduitis, which was the feature of chronic inflammation. In addition, 2 cases showed maternal infiltrating inflammatory cells in the subchorionic fibrin, but there was no evidence of acute villitis. As for the ascending intrauterine infection, placenta membrane examination showed that only 2 cases (case 4 and 5) presented neutrophils infiltration (more than 30 neutrophils per high-power field) in the fibrin-deposited fetal membrane tissues. However, neutrophils infiltration was limited to the fibrin under the chorionic lamina or the decidual layer of the fetal membrane, showing acute chorioamnionitis, maternal inflammatory response, stage 1 (acute chorionitis). Above of all, although a variety of inflammatory responses were identified in the placenta of pregnant women with SARS-CoV-2 infection, no typical changes such as massive mononuclear cell infiltration of the intervillous spaces and trophoblast necrosis which were considered as a risk factor for transplacental transmission of SARS-CoV-2 [19] were found in all eight cases. On the other hand, maternal vascular malperfusion (MVM) were present in all 8 cases. Features included central placental infarct (1/8), peripheral placental infarct (1/8), distal villous hypoplasia (1/8), increased syncytial knots (8/8, Fig. 1). Nomaternal decidual arteriopathy was noted in all cases. In addition, increased focal perivillous fibrin depositions were presented (7/8), and massive perivillous fibrin deposition pattern was observed (1/8). Impressively, both massive perivillous fibrin deposition pattern and obvious central placental infarct simultaneously were presented in the same area of case 2 (Fig. 1c) who had maternal hypertension, but this case did not appear to be accompanied by typical acute and chronic intervillositis. Moreover, no evidence of other maternal vascular disorders, such asplacental hematomas and floor infarction, were present in all placental tissues. No abnormalities were found in umbilical arteries and umbilical vein branches. There was no evidence of fetal vascular malperfusion (FVM).

**Table 2:** Pathological examination of the placental samples of the pregnant women with COVID-19 pneumonia

Pathological diagnosis	patient							
	1	2	3	4	5	6	7	8
**Category I: Maternal vascular malperfusion**								
Central placental infarct(s)		√						
Peripheral placental infarct			√					
Distal villous hypoplasia								√
Accelerated villous maturation pattern								
Increased syncytial knots	√	√	√	√	√	√	√	√
Villous agglutination								
**Category II:maternal decidual arteriopathy**								
Insufficient vessel remodelling								
Fibrinoid necrosis								
**Category III:Fetal vascular malperfusion (FVM)**								
Avascular fibrotic villi								
Thrombosis								
Intramural fibrin deposition								
Villous stromal-vascular karyorrhexis								
Stem villous vascular obliteration								
High-grade fetal vascular malperfusion								
**Category IV:Ascending intrauterine infection**								
Maternal inflammatory response (mild)				√	√			
Fetal inflammatory response								
**Category V:Fibrinoid**								
Increased focal perivillous fibrin depositions (perivillous fibrin plaque)		√	√	√	√	√	√	√
Massive perivillous fibrin deposition pattern		√						
Maternal floor infarct pattern								
**Category VI:Chronic inflammation**								
Chronic intervillositis								
Chronic plasma cell deciduitis						√		√
Chronic chorioamnionitis								
**Category VII:Other placental findngs**								
Microscopic accreta								
Villous edema								√
Membranes with hemorrhage								
Acute intervillositis (mild)								√

### Immuohistochemistry

**Fig. 2:**
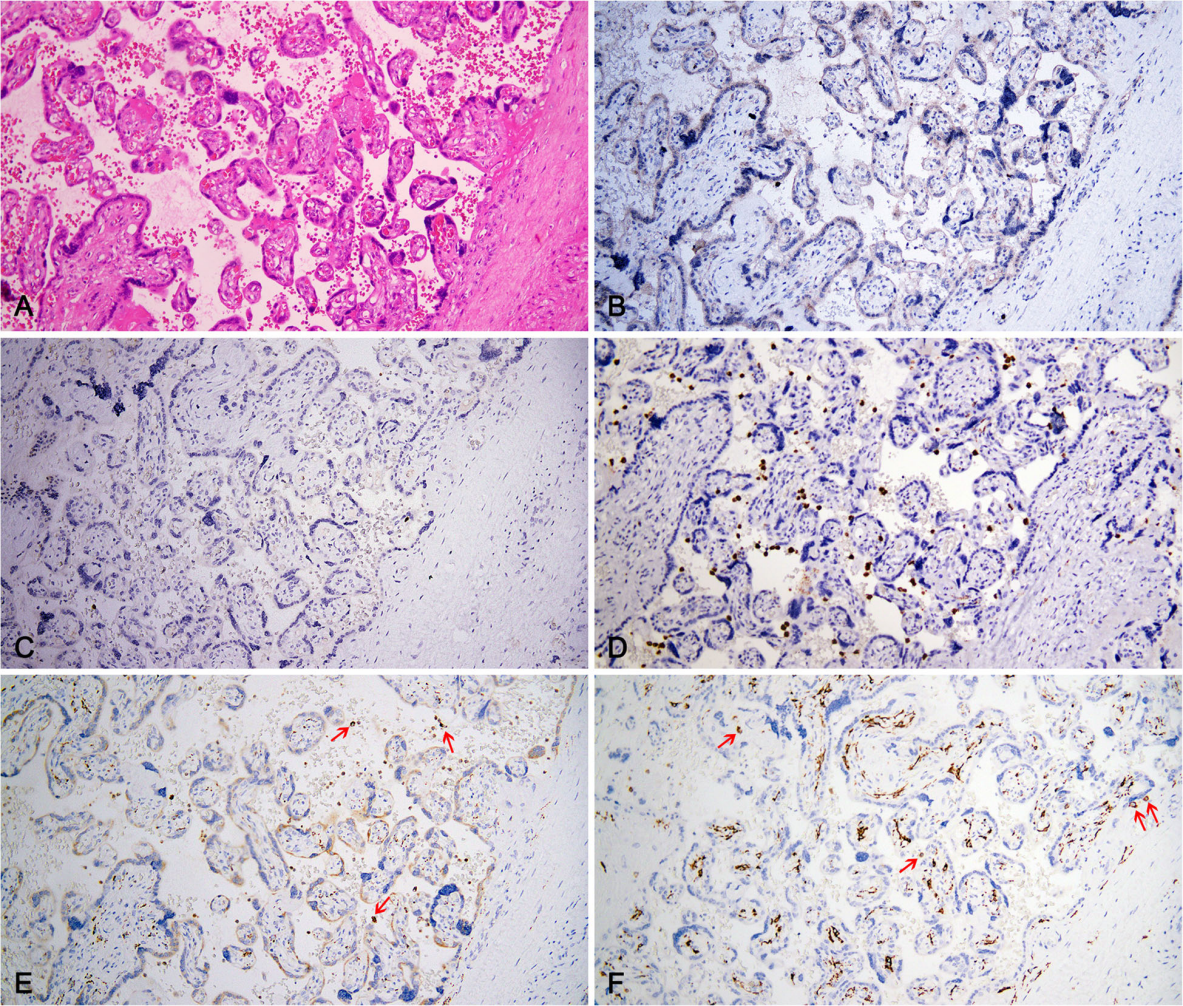
H&E and Immunohistochemical staining of inflammatory cells and Hofbauer cells of case 8. **a**, mild acute intervillositis was presented, the inflammatory infiltration is foci-like and consists of several neutrophils, no trophoblast necrosis and prominent perivillous fibrin deposition was found in the intervillous space. **b and c** CD3 (**b**) and CD20 (**c**) staining revealed only occasional infiltration of T lymphocytes and B lymphocytes in the middle of terminal villi (Envision, Original magnifications × 200). **d,** CD15 staining revealed several neutrophils in the intervillous space. **e** and **f,** CD68 (**e**) and CD163 (**f**) revealed scanty histiocytes in the intervillous space (red arrows) and none prominently increased numbers of Hofbauer cells present in the stroma of all villi (Envision, original magnifications × 200)

**Fig. 3:**
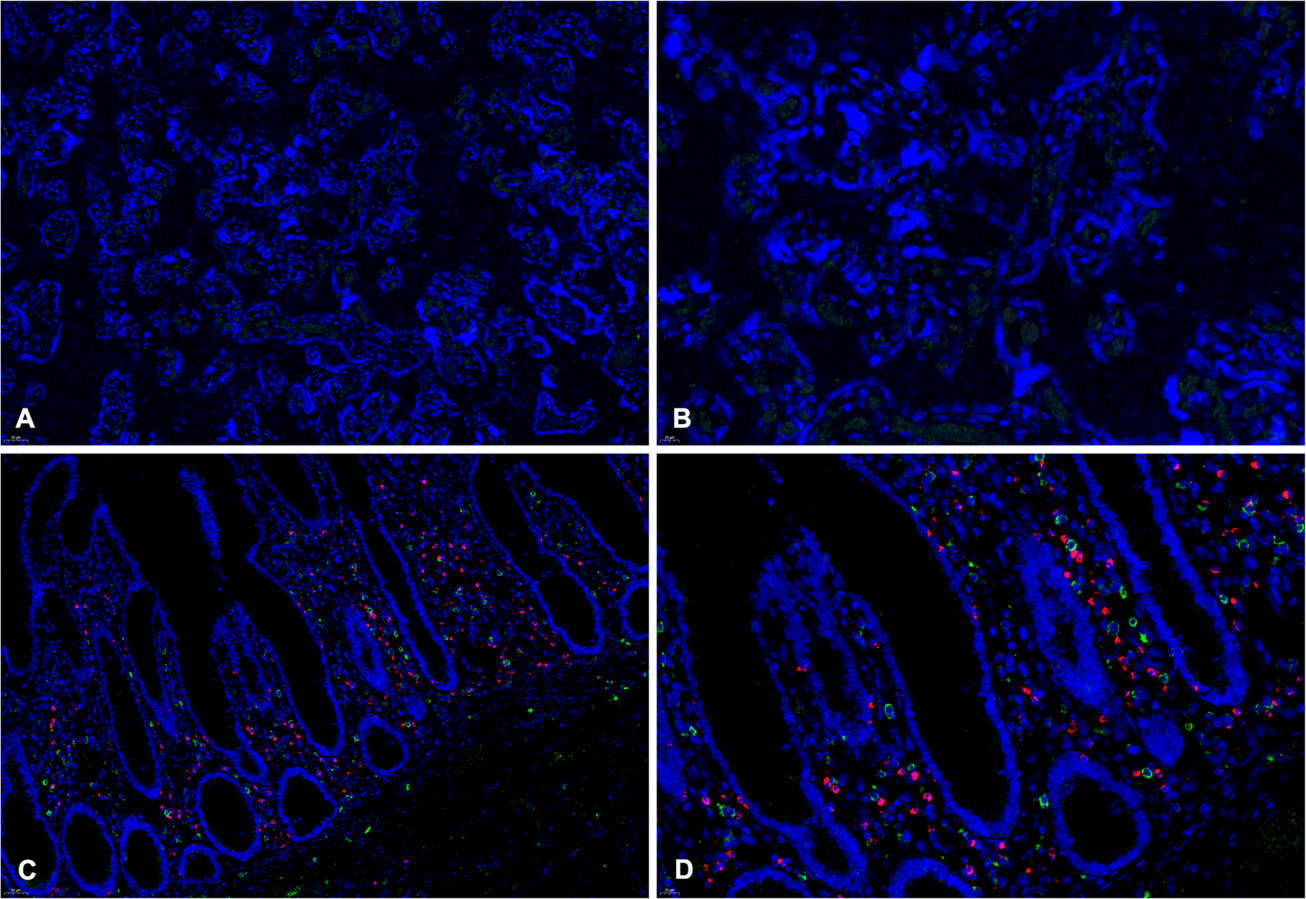
IF and FISH double staining result of SARS-CoV spike protein (green) and SARS-CoV-2 RNA (red) in all eight cases and positive control. **a**, Original magnifications × 200) and **b**, Original magnifications × 400):no positive signal was presented of monoclonal antibody against SARS-CoV spike protein (green) and SARS-CoV-2 RNA (red) in all eight cases by IF and FISH double staining. **c**, Original magnifications × 200) and **d**, Original magnifications × 400): both of positive signal were presented in acolon tissue with COVID-19 infection as positive control

In case 8, several neutrophils and scanty histiocytes were seen in the intervillous space, which were confirmed by CD15,CD163 and CD68 positive staining (Fig. 2). In the other seven cases, there was no significant increase in the staining of macrophages in placental villous and decidual tissues of the third trimester, as reflected by the positive staining of CD163 and CD68. In addition, there was no increase in the number of inflammatory cells such as T cells, B cells and plasma cells in all placental villi tissues, while the quantity of T cells in the decidua tissue of 2 patients (case 4 and 5) slightly increased. And 2 cases who had chronic plasma cell deciduitis (case 5 and 7) showed that the quantity of plasma cells in the decidua tissues has increased significantly. Moreover, no positive result of a monoclonal antibody against SARS-CoV spike protein was presented in all eight cases (Fig. 3), contrasted with positive signal in the colon tissues of a COVID-19 patient as positive control by IF.

### Viral RNA detection

The presence of SARS-CoV-2 RNA was detected in the placenta tissue samples collected from eight patients. All of eight cases demonstrated negative results by FISH using a SARS-CoV-2 virus RNA probe compared with positive control presented in a colon tissue with COVID-19 infection (Fig. 3).

## Discusion

This study retrospectively analyzed the clinical characteristics of 8 cases of COVID-19 pregnant women in third trimester. The limited data showed that the clinical manifestations of SARS-CoV-2 infected pregnant women were basically similar to those of the general infected population, and there were no serious adverse mother-infant outcomes. In this study we performed microscopic observations and immunohistochemical tests at the same time to determine whether the number of inflammatory cells and fetal-derived placental macrophages (Hofbauer cells) in the placenta from pregnant women with COVID-19 has increased. No specific inflammatory pathological changes suggesting SARS-CoV-2 invasion of the placenta were present through the microscopic observation and IHC detection. FISH detection of SARS-CoV-2 RNA in placental tissues and RT-PCR detection of neonatal pharyngeal swabs in all cases were negative. This study suggested no definite evidence pointing to maternal-fetal vertical transmission in pregnant women with COVID-19 in late pregnancy, and provided important clues for further understanding of the clinical characteristics, pregnancy outcomes, and evaluation of intrauterine transmission of SARS-CoV-2 infection in late pregnancy.

The main histopathological features of placenta viral infection which occurred in some TORCH agents [26], such as cytomegalovirus, *Treponema pallidum*, *Toxoplasma*, and rubella virus infection and other hematogenously transmitted infections through the placenta [27] showed significant inflammatory abnormalities, such as chronic villitis, intervillositis, and funisitis. The existing limited placental pathology studies related to SARS-CoV-2 placental infection [13–15, 18] suggested that significant chronic histiocytic intervillositis often be appeared, and the feature has been regarded as a risk factor for COVID-19 placental infection and mother-to-child transmission [19]. Fortunately, in our study, no typical chronic histiocytic intervillositis was found in all eight placental tissues and no evidence of worse maternal disease were present. Although in our study individual cases showed corresponding inflammation changes in the placental tissues, such as maternal inflammatory response (mild), mild acute intervillositis and chronic plasma cell deciduitis, which indicated there might be other pathogen infections needs further study. This phenomenon was consistent with the results of the existing limited studies [28, 29]. In addition, as several other studies on placental pathology [13–15, 18], we also performed IHC analysis on inflammatory cells, especially Hofbauer cells, in late placenta tissue from COVID-19 pregnant women. As Hofbauer cells can harbor live virus such as ZIKA virus [22], HIV virus [30] and Cytomegalo virus [31]and serve as reservoirs within the placenta, it is one of the important ways to transmit pathogens to fetal-placenta tissues by infecting Hofbauer cell [32, 33]. However, H&E staining and IHC showed no significant infiltration of T cell or evidence of villous stromal macrophages hyperplasia. FISH analysis further enhanced the evidence that no virus directly infected the placenta, which was similar to the results of previous limited studies [28, 34].

On the other hand, the stage of gestation at the time of infection may affect whether SARS-CoV-2 virus was vertical transmission. Stage of gestation has been proved as an important factor affecting the mechanisms of maternal-fetal vertical transmission [35]. For example, in the early infection of rubella virus, more than 50% of fetuses were infected vertically through the uterus, but as the pregnancy time increases, the risk of vertical transmission was significantly reduced [36]. The phenomenon was also present in the ZIKA virus. Since higher ZIKA virus titers were detected in amniotic epithelial cells from mid-gestation, suggesting a greater susceptibility of virus infection in the placenta from the second trimester or earlier compared to late-gestation placentas [16]. As with previous studies [28, 37, 38], our study mainly included pregnancy women with infection in the third trimester and found no evidence of vertical transmission, further suggesting that the placenta may play a greater and powerful barrier role to prevent SARS-CoV-2 infection in the third trimester, and the specific resistance mechanism still needs to be further studied.

Although the defense mechanism of placenta to restrict microorganisms from entering the fetus is largely unclear, existing evidence suggested that syncytiotrophoblasts can effectively resist numerous pathogens, and cytotrophoblasts also has an innate defense mechanism against intracellular pathogens [16]. Impressively, the syncytiotrophoblast layer has strong resistance to various viruses such as HCMV, HSV1, and ZIKA [39–41] in the late pregnancy. For example, trophoblasts are sensitive to ZIKA virus at the earliest stage of trophoblast development, but become more and more resistant when the syncytium forms in late pregnancy [42]. So whether trophoblasts play a part in the mechanism of placental resistance of the SARS-CoV-2 virus in late pregnancy will be the direction of our further research.

Notably, another most striking observation in the placentas (all 8 cases) was the prominent and diffuse increase of syncytial knots, which was one of the features of MVM. As first described by Tenney and Parker [43], syncytial knots were the aggregations of syncytiotrophoblast nuclei, and their increase may involve nearly all terminal villi in preeclampsia, whereas they were only appeared in 10–15% normal terminal villi [44].Moreover, exposure of the placenta to conditions such as hypoxia, hyperoxemia, or oxidative stress may cause an increase in syncytial knots [45].And our results were consistent with the existing evidence on the pathology of placentas with coronavirus infection, which exhibited a few abnormalities about MVM [28, 46], such as increased syncytial knots, different degrees of fibrin deposition in intervillous and subchorion, which could also be observed in this study. Given that all cases collected in this study were asymptomatic or with mild syndrome, so the results suggested that mild symptoms of SARS-CoV-2 infection might induce the decline in oxygenation within the intervillous space and cause a degree of placental injury, although there was no clear evidence of SARS-CoV-2 infection of the placenta in the third trimester. This is of great significance to the safety of mothers and fetuses in late pregnancy.

Consistent with several recent case reports [13, 18], FISH was performed to detect SARS-CoV-2 RNA in the placenta, but no evidence of SARS-CoV-2 invasion in the late gestation placenta was present in our study. Although the recent case report suggested the presence of SARS-CoV-2 in 3/11 swabs of the placenta or membrane by RT-PCR [47], swab samples rather than tissue samples of the placenta or membranes might increase the possibility of virus droplet contamination in the hospital environment or virus exposure during delivery, so they could not be used as direct evidence of vertical transmission. In addition, recently, several studies [12, 15, 18, 48, 49] also have demonstrated the presence of the SARS-CoV-2 virus by RT-PCR in the placenta tissues. However, as the placenta biopsy or tissue sample included two different cells of both maternal and fetal origin cells, therefore, the positive result of the placenta sample tested by RT-PCR cannot be used to assess whether the SARS-CoV-2 virus came from the mother or the fetus [20]. Compared with RT-PCR, FISH analysis directly used tissue samples for detection, which displayed the precise cell location of fusion genes and relevant information on the anatomical distribution of the placenta [33]and helped to provide clues for exploring the mechanism of placental virus infection or defense. Above all, it can be seen that FISH is practicable and can provide more information to diagnosis SARS-CoV-2 invasion of the placenta.

This study still has some limitations. First of all, the cases collected in this study were all mild patients, and it was still unknown whether patients with severe infections in pregnancy will develop intrauterine infection, which is the direction for further research in later research. Secondly, a recent report suggested that positive SARS-CoV-2 infection in the second trimester pregnancy women can lead to miscarriage, and the evidence of SARS-CoV-2 infection in the placenta had also been found [18]. So further cases including different gestation stage women of COVID-19, especially in the first and second trimester, need to be collected to study the effect to maternal and fetus safety.

In summary, we found no evidence of vertical transmission in the third trimester placenta of COVID-19 pregnancy women by observing histological changes and nucleic acid test, we also analyzed whether the number of the inflammatory cells and macrophages cells increased by immunohistochemistry. Although the sample size of this study was limited, considering the important adverse effects of this ongoing global public health emergency, our results were very useful for understanding the clinical characteristics of COVID-19 infection in late-stage pregnant women and whether it has the potential for vertical transmission. It was important and provided a certain basis for the best clinical management of late pregnant women.

## Data Availability

All data generated or analysed during this study are included in this published article.

## Abbreviations

CoV: Coronavirus
COVID-19: 2019 novel coronavirus (CoV) disease
SARS: Severe acute respiratory syndrome
FISH: Fluorescence In-Situ Hybridization
IF: Immunofluorescence
MVM: Maternal vascular malperfusion
SARS-CoV-2: Severe acute respiratory syndrome coronavirus 2
MERS: Middle East respiratory syndrome
NP: Nasopharyngeal
RT-PCR: Real-time reverse transcription polymerase chain reaction
IHC: Immunohistochemistry
FFPE: Formalin-fixed, paraffin-embedded
ALT: Alanine aminotransaminase
AST: Aspartate aminotransferase
FVM: Fetal vascular malperfusion
